# Exosome-mediated immunomodulatory and barrier-protective responses of adipose-derived mesenchymal stem cells in oleic acid-induced acute lung injury

**DOI:** 10.1590/acb416926

**Published:** 2026-10-19

**Authors:** Gamze Ayar, Badel Ince, Kansu Buyukafsar, Leyla Bahar, Elif Onsoz, Erkan Maytalman, Mehmet Oguz Koksel

**Affiliations:** 1 Mersin University – Institute of Health Sciences – Department of Stem Cell and Regenerative Medicine – Mersin – Turkey.; 2 Mersin University – Faculty of Medicine – Department of Medical Pharmacology – Mersin – Turkey.; 3 Alanya Alaaddin Keykubat University – Faculty of Medicine – Department of Medical Pharmacology – Antalya – Turkey.; 4 Mersin University – Faculty of Medicine – Department of Thoracic Surgery – Mersin – Turkey.

**Keywords:** Respiratory Distress Syndrome, Acute Lung Injury, Mesenchymal Stem Cells, Exosomes, Regenerative Medicine

## Abstract

**Purpose:**

Acute lung injury (ALI) is a severe pulmonary disorder with limited treatment options. Mesenchymal stem cell-based therapies show regenerative and immunomodulatory potential, but the comparative efficacy of adipose-derived mesenchymal stem cells (ADMSCs) and their exosomes remains unclear. This study evaluated their therapeutic effects in an experimental ALI model.

**Methods:**

ADMSCs were isolated from rat adipose tissue and cultured under standard conditions. Exosomes were characterized by flow cytometry (CD9, CD63, CD81), scanning electron microscopy, dynamic light scattering, and zeta potential analysis. ALI was induced in Wistar Albino rats by intravenous administration of 60 mg/kg oleic acid. Treatments were administered 2.5 hours after injury. Lung tissues were examined histopathologically after 24 hours, and mRNA expression levels of interleukin (IL)-6, IL-10, and vascular endothelial growth factor (VEGF) were analyzed.

**Results:**

Both ADMSC and exosome treatments significantly reduced lung injury scores compared with the oleic acid group. ADMSCs demonstrated greater histopathological improvement than exosomes, although no significant differences were observed in IL-6, IL-10, or VEGF expression.

**Conclusion:**

Both ADMSCs and their exosomes showed therapeutic potential in ALI, with stem cells providing superior histopathological recovery.

## Introduction

Acute lung injury (ALI) and acute respiratory distress syndrome (ARDS) are critical respiratory conditions characterized by high mortality rates and limited therapeutic options^1^. Patients frequently require mechanical ventilation due to impaired alveolar gas exchange, pulmonary edema, and intense neutrophilic inflammation^1,2^. Given the global burden and the lack of effective pharmacological strategies, discovering novel therapeutic approaches to accelerate pulmonary recovery remains a pressing need.

Experimental animal models are essential for investigating ALI pathophysiology and evaluating potential interventions. Among them, the oleic acid-induced model is widely utilized due to its ability to successfully reproduce key clinical and histopathological features of human ALI, particularly by replicating acute endothelial cell alterations and associated vascular permeability changes^3^.

Recently, cell-based and cell-free regenerative strategies have emerged as promising alternatives. Adipose-derived mesenchymal stem cells (ADMSCs) offer strong paracrine and immunomodulatory effects, showing beneficial outcomes in various lung injury models^4-7^. Similarly, ADMSC-derived exosomes extracellular vesicles carrying bioactive molecules have gained interest as biocompatible, cell-free therapeutic agents capable of modulating tissue repair without the risks associated with whole-cell transplantation^8^. Both interventions are thought to influence key molecular mediators of inflammation and tissue remodeling, such as interleukin (IL)-6, IL-10, and vascular endothelial growth factor (VEGF)^9-14^.

Despite the documented regenerative potential of ADMSCs and their exosomes, a direct comparison of their therapeutic efficacy specifically within an oleic acid-induced ALI model remains poorly explored. Therefore, the purpose of this study was to compare the histopathological and molecular effects of ADMSCs and ADMSC-derived exosomes in a Wistar albino rat model of oleic acid-induced ALI. We investigated lung tissue regeneration and monitored the sequential alterations in mRNA expression levels of IL-6, IL-10, and VEGF following early therapeutic intervention.

## Methods

A schematic overview of the experimental workflow and the chronological sequence of the study procedures is presented in Fig. 1.

**Figure 1:**
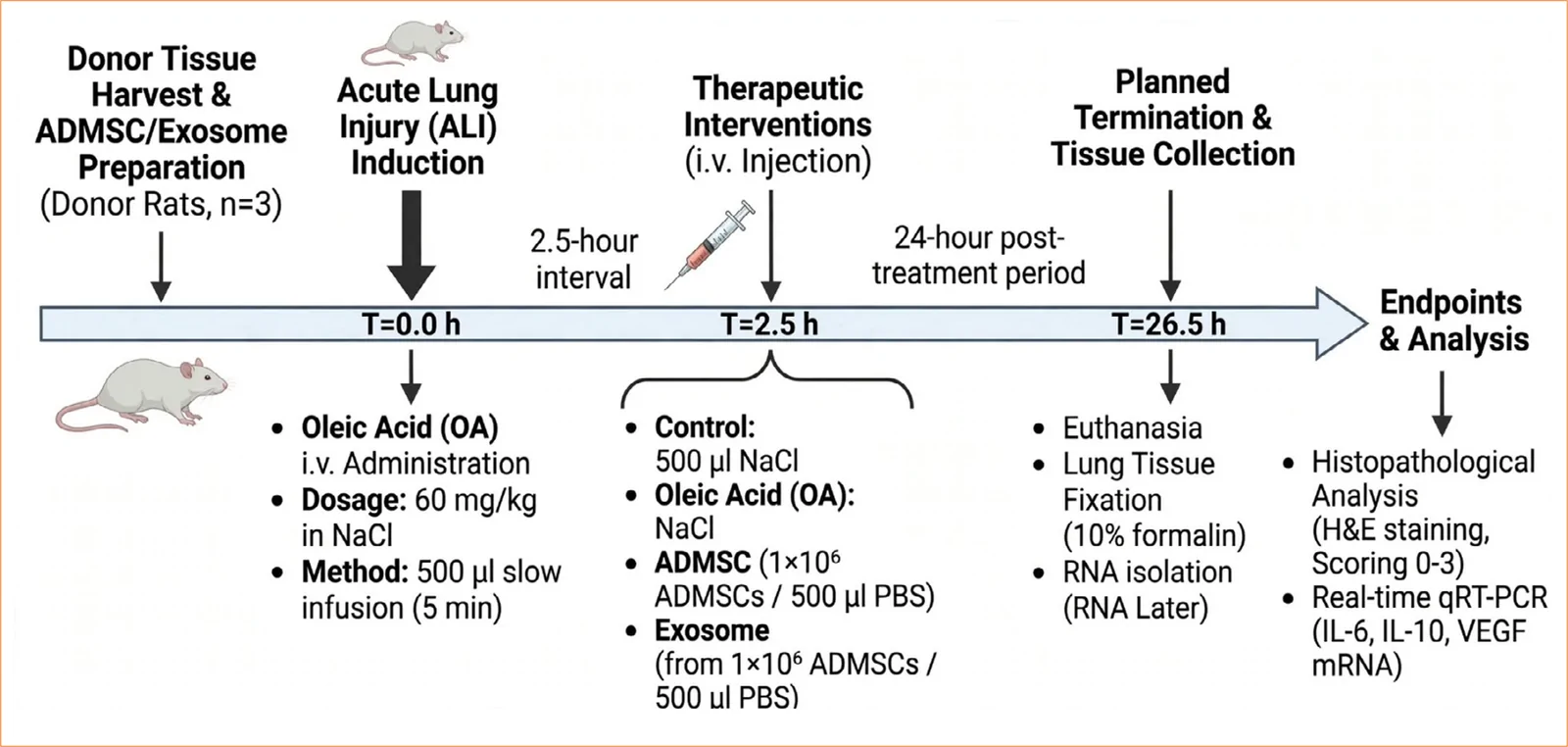
Schematic timeline of the experimental study design. The timeline illustrates the chronological sequence of the major experimental procedures, from animal allocation and experimental preparation to treatment, tissue collection, and subsquent analyses.

ADMSC: adipose-derived mesenchymal stem cell; PBS: phosphate buffered saline; H&E: hematoxylin and eosin; qRT-PCR: quantitative real-time polymerase chain reaction; IL: interleukin; VEGF: vascular endothelial growth factor. Source: Elaborated by the authors.

### Ethical approval and experimental animals

This study was conducted in accordance with the Guide for the Care and Use of Laboratory Animals and was approved by the Mersin University Center for Experimental Medicine. All procedures complied with the ethical guidelines of the Mersin University Experimental Animals Research Unit (ethics committee approval number: 2021/HADYEK/34).

A total of 24 mature male Wistar Albino rats weighing 220–250 g were used for the experimental procedures. All animals were housed under identical environmental conditions, including controlled temperature, humidity, and light–dark cycle, and were provided the same diet and water *ad libitum*.

For the preparation of ADMSCs, three additional mature male Wistar Albino rats were used exclusively as donor animals. These three donor rats were not included in the four experimental groups and were used only for adipose tissue collection and subsequent ADMSC isolation.

### Experimental design, randomization, and group allocation

The 24 experimental rats were randomly allocated to four experimental groups (n = 6 per group). To achieve balanced distributions of age and body weight across the groups, an optimization procedure based on the Nelder–Mead algorithm was used. Animals from different experimental groups were evenly distributed among the cages to minimize potential cage-related confounding effects, and cage positions were rotated regularly throughout the experiment.

All treatments and sample collections were performed within the same daily timeframe to minimize potential circadian effects. In addition, the order of procedures, including injections, euthanasia, and tissue collection, was randomized daily to reduce systematic bias related to time-dependent factors.

The experimental groups were as follows:

- Control group: received 500 µL of NaCl solution intravenously;
- Oleic acid (OA) group: received 500 µL of a solution containing NaCl and 60 mg/kg oleic acid intravenously;
- Adipose-derived mesenchymal stem cell treatment (ASCT) group: received 1 × 10^6^ ADMSCs suspended in 500 µL phosphate buffered saline (PBS) intravenously 2.5 h after OA administration.
- Exosome treatment (EXT) group: received exosomes isolated from 1 × 10^6^ ADMSCs, suspended in 500 µL PBS intravenously 2.5 h after OA administration.

Humane endpoints were established to minimize animal suffering. Animals were monitored twice daily for clinical signs, including labored breathing, lethargy, persistent recumbency, and refusal to eat or drink. No animals met the predefined humane endpoint criteria, and all experimental animals survived until the planned termination point.

### Isolation and culture of adipose-derived mesenchymal stem cells

Abdominal and epididymal adipose tissues (10–20 g) were harvested from the three additional donor rats following euthanasia. The collected tissues were placed in PBS supplemented with 5% penicillin–streptomycin and incubated for 1 h.

The tissues were subsequently enzymatically digested with type I collagenase at 37°C for 40 min. Following enzymatic digestion, the cell suspensions were washed with PBS, and the resulting cell pellets were resuspended in AlphaMEM culture medium containing 10% fetal bovine serum, 2 mM of L-glutamine, 100 IU/mL of penicillin, and 100 µg/mL of streptomycin.

The cell suspensions were seeded into T25 cm^2^ culture flasks at a density of 1 × 10^6^ cells/mL. The cells were maintained until they reached 85–90% confluency. After seven days, the cells were passaged, and cells at the third passage were collected and used for the experimental procedures.

### Isolation of adipose-derived mesenchymal stem cell-derived exosomes

At the third passage, ADMSCs were cultured in AlphaMEM supplemented with 10% exosome-depleted fetal bovine serum (catalog number: A2720801, Thermo Fisher Scientific, United States of America). Exosomes were isolated from the conditioned cell culture medium using the total exosome isolation kit (catalog number: 4478359, Life Technologies, Carlsbad, CA, United States of America), according to the manufacturer’s instructions.

The isolated exosomes were subsequently characterized before their use in the exosome treatment group.

### Characterization of adipose-derived mesenchymal stem cell-derived exosomes

#### Flow cytometry analysis

The isolated exosomes were characterized by flow cytometry based on the expression of the exosomal surface markers CD9, CD63, and CD81 using the MACSPlex exosome kit (catalog number: 130122209, Miltenyi Biotec, Germany), according to the manufacturer’s instructions.

### Scanning electron microscopy analysis

For scanning electron microscopy (SEM) analysis, a 25-µL droplet of the exosome suspension in 1× PBS was placed onto a glass coverslip and allowed to dry for 4 h at room temperature, following a methodology modified from a previously described literature.^15^ The samples were subsequently coated with a platinum–palladium layer and systematically imaged using a Carl Zeiss SUPRA 55VP field-emission SEM at an accelerating voltage of 5 kV.

### Dynamic light scattering and zeta potential analysis

The hydrodynamic diameter of the isolated exosomes was determined by dynamic light scattering (DLS) using a Zetasizer Nano system (Malvern Panalytical Ltd., Malvern, United Kingdom). DLS measures the Brownian motion of particles in suspension by detecting fluctuations in the intensity of scattered laser light. In addition to particle size, the surface charge of the exosomes was evaluated by measuring the zeta potential, which provides information regarding their colloidal stability.

### Oleic acid-induced acute lung injury and administration of adipose-derived mesenchymal stem cell and exosomes

ALI was induced using OA. A 13% OA stock solution was prepared by dissolving 5 g of oleic acid in 10 mL of ethanol. The volume required for each animal was calculated according to individual body weight and subsequently diluted with NaCl to obtain the final injection volume.

Lung injury was induced by intravenous administration of 500 µL of the diluted OA solution at a constant flow rate over 5 min. Control animals received 500 µL of NaCl solution intravenously.

Following OA administration, the animals were monitored for 2.5 h. At the end of this period, the ASCT group received 1 × 10^6^ ADMSCs suspended in 500 µL PBS intravenously, whereas the EXT group received exosomes isolated from 1 × 10^6^ ADMSCs and suspended in 500-µL PBS. Treatments were administered intravenously using an insulin syringe according to the predefined experimental group assignments. The OA group did not receive ADMSC or exosome treatment.

### Tissue collection and histopathological analysis

Twenty-four hours after the administration of ADMSCs or exosomes, the animals were euthanized, and lung tissue samples were collected for histopathological and molecular analyses.

For histopathological analysis, lung tissue samples were fixed in 10% formalin. Tissue blocks were sectioned at a thickness of 4–5 µm using a microtome and subsequently processed through serial xylene steps. Following dehydration, the sections were stained with hematoxylin and eosin (H&E).

Lung injury was evaluated using a standardized scoring system, with scores ranging from 0 to 3 based on the degree of inflammatory cell infiltration, edema, hemorrhage, and hyperplasia.

### Molecular analysis of inteleukin (IL)-6, IL-10, and vascular endothelial growth factor expression

Molecular analyses were performed to evaluate the responses to ADMSC and exosome treatments in the context of OA-induced ALI. IL-6 and IL-10 were selected as representative pro-inflammatory and anti-inflammatory cytokines, respectively, whereas VEGF was included as a marker of angiogenesis. Quantitative real-time polymerase chain reaction (qRT-PCR) was used to determine the relative mRNA expression levels of IL-6, IL-10, and VEGF in lung tissue samples obtained from the experimental groups.

### Total RNA isolation

For RNA extraction, 50 mg of lung tissue was collected into sterile tubes containing RNA Later solution and homogenized using a plastic mallet. Total RNA was isolated using the GeneAll RiboEx kit (catalog number: 301-001, GeneAll, South Korea) and Hybrid-R kit (catalog number: 305-101, GeneAll, South Korea), following the manufacturers’ protocols.

### cDNA Synthesis

RNA concentrations were quantified using a fluorometric method. Following optimization of RNA concentrations, cDNA was synthesized using the WizScript cDNA synthesis kit (catalog number: W2201, Wizbio Solutions, Korea), according to the manufacturer’s instructions.

### Real-time polymerase chain reaction

Real-time PCR reactions were prepared using the WizPure^TM^ qPCR Master (SYBR) kit (catalog number: W1711, Wizbio Solutions, South Korea), according to the manufacturer’s protocol. Amplification was performed using an Applied Biosystems^TM^ 7500 Fast Real-Time PCR system.

Relative expression levels of IL-6, IL-10, and VEGF were calculated using the 2^−ΔΔCt^ method. The RNA expression levels of each sample were normalized to those of the control group, and glyceraldehyde 3-phosphate dehydrogenase (GAPDH) was used as the reference gene. Real-time PCR reactions were performed in triplicate for each sample. The primer sequences used for the target genes are presented in Table 1.

**Table 1:** Primer sequences of interleukin (IL)-6, IL-10 and vascular endothelial growth factor (VEGF) genes.

Primer name	Primer sequences
IL-6	F: AACAAGAAAGACAAAGCCAGAGT R: TTGGGGTAGGAAGGACTATTTTA
IL-10	F: ATGTTGCCTGCTCTTACTGG R: TCCACTGCCTTGCTTTTATTC
VEGF	F: CAATGATGAAGCCCTGGAGT R: TTTGACCCTTTCCCTTTCCT
*GAPDH	F: AGAACATCATCCCTGCATCC R: GTCCTCAGTGTAGCCCAGGA

* Glyceraldehyde 3-phosphate dehydrogenase (GAPDH) gene expression level was determined as an endogenous control gene.

Source: Elaborated by the authors.

### Statistical analysis

The normality of the data was assessed using the Shapiro–Wilk’s test, and the homogeneity of variances was evaluated using Levene’s test. The histopathological total lung injury scores and mRNA expression values for IL-6, IL-10, and VEGF were normally distributed and showed homogeneous variances.

Accordingly, differences among the experimental groups were evaluated using one-way analysis of variance (ANOVA). Tukey’s post hoc test was used for pairwise subgroup comparisons. All statistical analyses were performed using Statistical Package for the Social Sciences Statistics version 21. A *p*-value < 0.05 was considered statistically significant.

## Results

The therapeutic effects of ADMSCs and their exosomes were evaluated in a rat model of oleic acid-induced ALI with histopathological analysis and mRNA expression profiling. Lung tissue samples were assessed to determine the extent of injury and inflammation, while the mRNA expression levels of IL-6, IL-10, and VEGF were measured to investigate the molecular responses associated with inflammation and tissue regeneration.

### Flow cytometry analysis of exosomes derived from adipose-derived mesenchymal stem cell

Exosomes derived from ADMSCs were characterized by flow cytometry (Fig. 2). Figure 2a depicts the exosome profile in the allophycocyanin and phycoerythrin channels, where 100 represents the negative control and 101 represents the exosome population. Analysis revealed distinct positive populations for the exosomal markers CD9, CD63, and CD81 in exosomes. Among these markers, CD63 exhibited the highest signal intensity, while CD81 and CD9 showed comparatively moderate and lower expression levels, respectively. The analysis revealed that the isolated exosomes comprised 58.28% of the total population (Fig. 2b).

**Figure 2:**
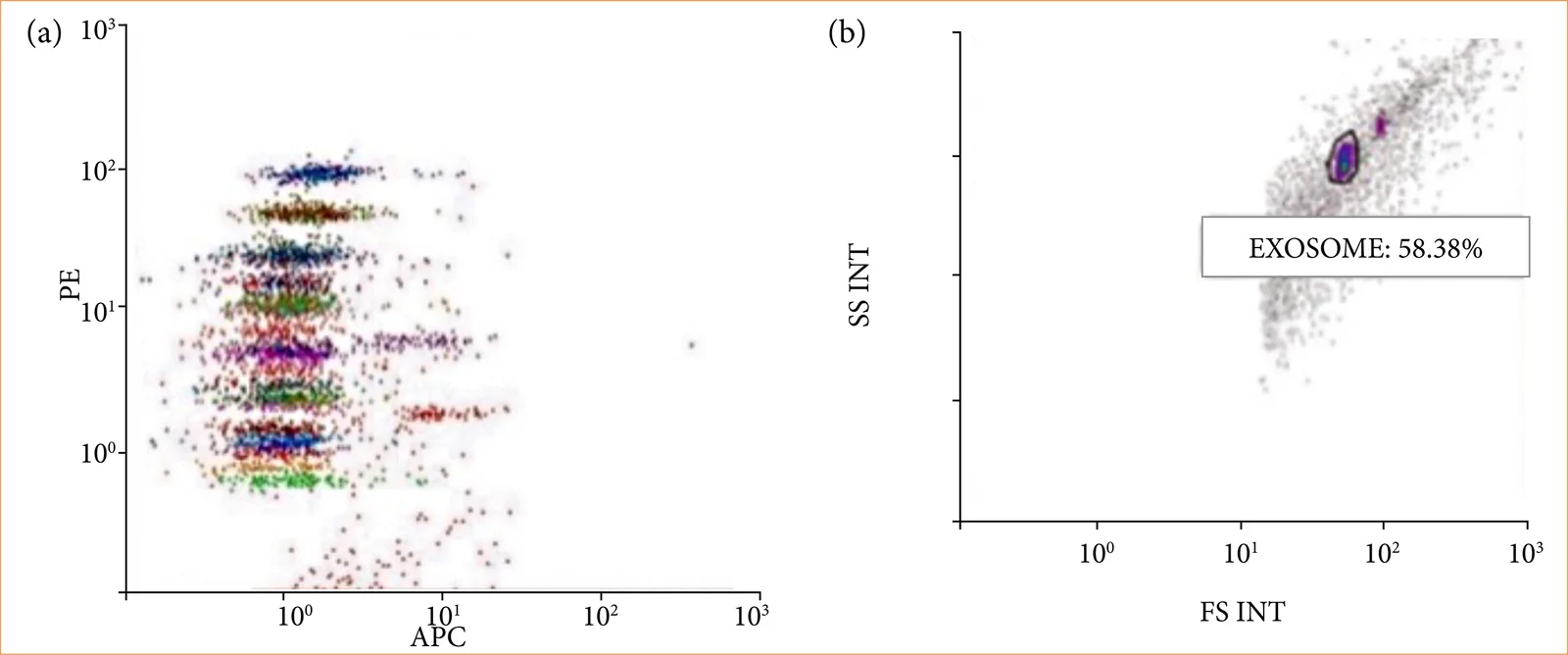
Flow cytometry analysis of exosomes derived from adipose-derived mesenchymal stem cells. (a) Exosome profile in the allophycocyanin and phycoerythrin channels: 10^0^ represents the negative control and 10^1^ the exosome population. (b) Isolated exosomes comprised 58.28% of the total population.

Source: Elaborated by the authors.

### Scanning electron microscopy analysis of exosomes derived from adipose-derived mesenchymal stem cell

SEM imaging showed that the isolated exosomes had a homogeneous population of nano-sized, spherical to cup-shaped structures, supporting the successful isolation of exosomes (Figs. 3 and 4).

**Figure 3:**
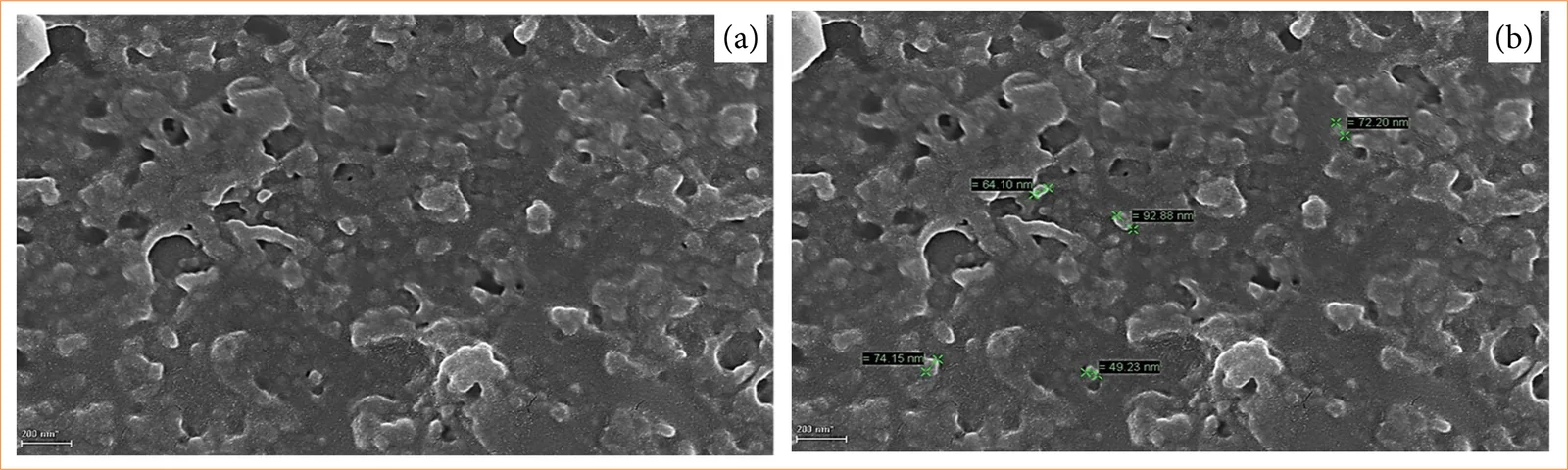
Scanning electron microscopy. (a) Scanning electron microscopy image at 100× magnification of exosomes (scale bar: 20 nm). (b) Scanning electron microscopy image at 100× magnification showing size measurements of individual exosomes (scale bar: 200 nm).

Source: Elaborated by the authors.

**Figure 4:**
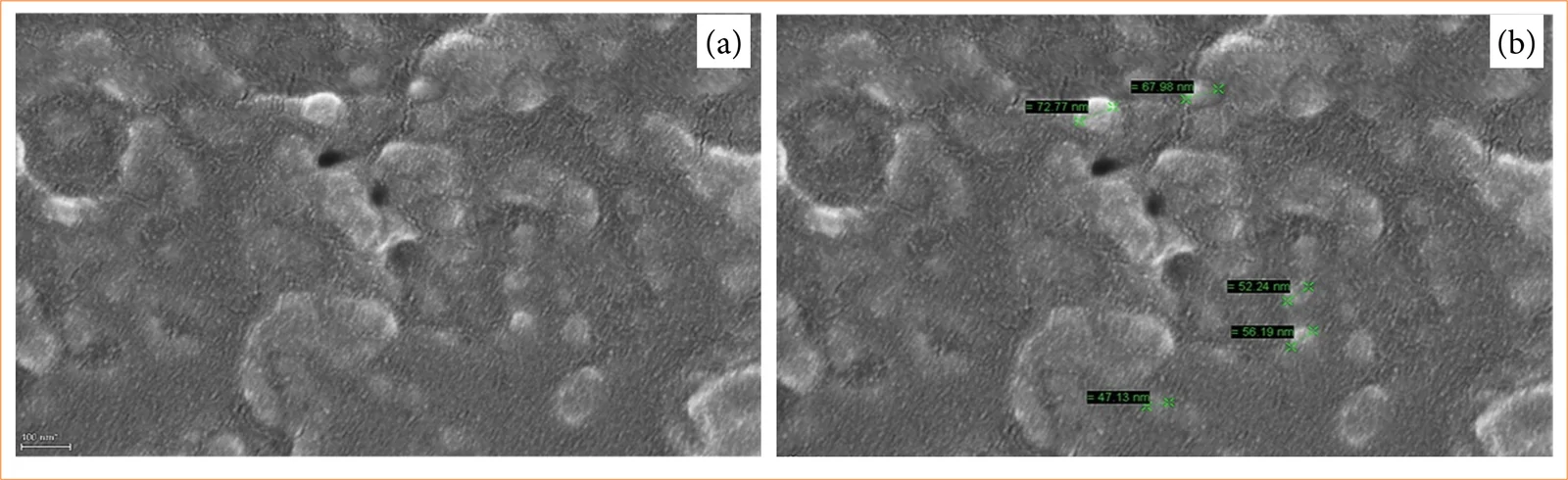
Scanning electron microscopy images of exosomes. (a) Scanning electron microscopy image at 200× magnification of exosomes (scale bar: 100 nm). (b) Scanning electron microscopy image at 200× magnification showing size measurements of individual exosomes (scale bar: 100 nm).

Source: Elaborated by the authors.

### Dynamic light scattering and zeta analysis of exosomes derived from adipose-derived mesenchymal stem cell

DLS analysis revealed an intensity-based Z-average hydrodynamic diameter of 89.34 nm with a polydispersity index of 0.288, indicating a moderately homogeneous vesicle population (Fig. 5). Volume-weighted size distribution analysis showed a dominant particle population with a peak diameter of 69.8 nm. Number-weighted size distribution indicated a predominant population centered at 18.6 ± 9.3 nm. Zeta potential measurements revealed that the exosome samples had an overall negative surface charge, with values ranging from -4.13 to -20 mV. The average zeta potential was -9.3 ± 5.5 mV at 25°C, suggesting a low-to-moderate colloidal stability of the vesicle suspension (Table 2).

**Figure 5:**
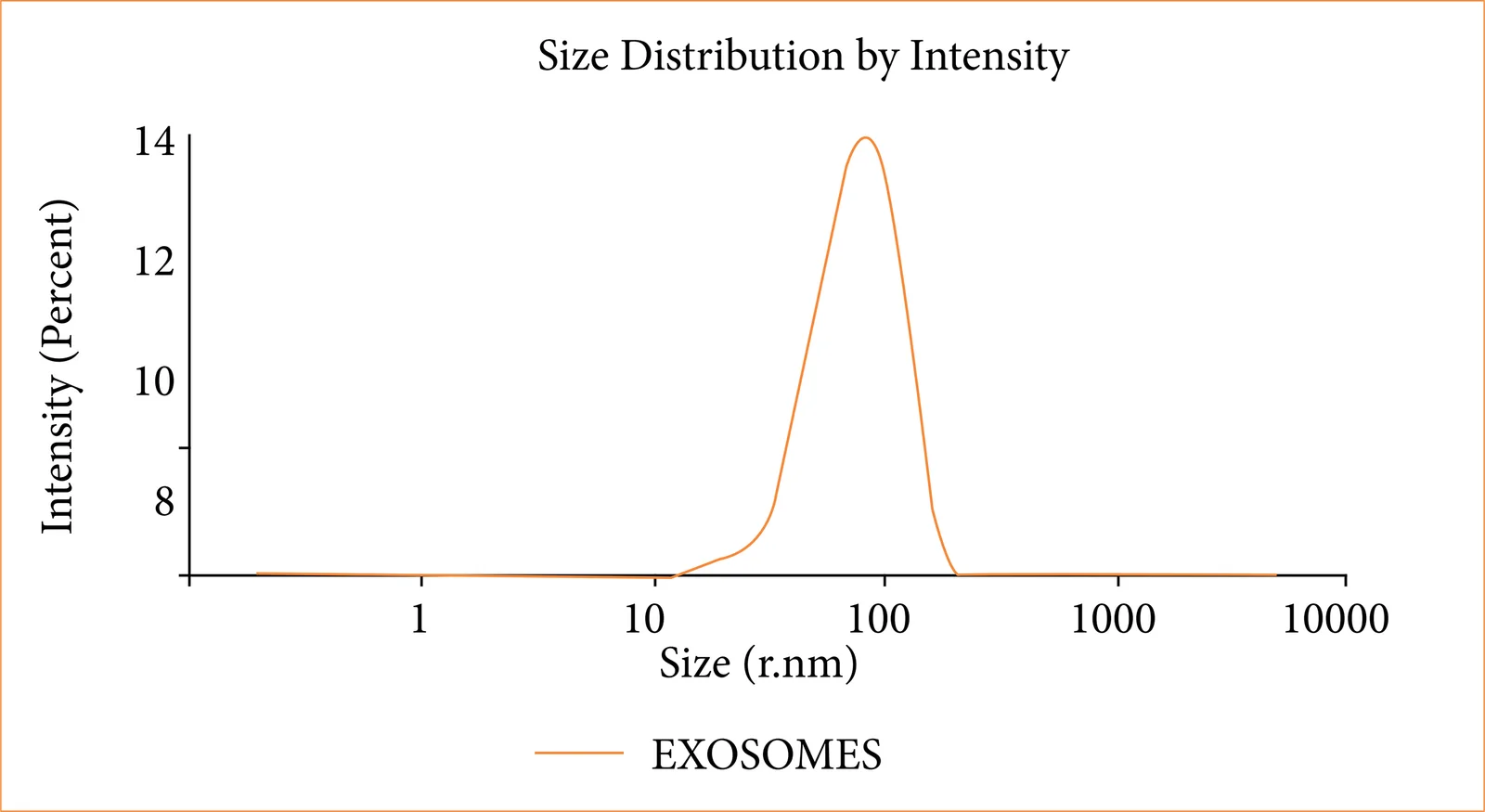
Intensity-based size distribution of isolated exosomes obtained by dynamic light scattering.

Source: Elaborated by the authors.

**Table 2:** Dynamic light scattering and zeta analysis results of exosomes.

	Z-average (nm)	PdI	Volume peak (nm)	Number mean (nm)	Zeta potential (mV)
Exosomes	89.34	0.288	~69.8	18.6 ± 9.3	-9.3 ± 5.5

Source: Elaborated by the authors.

### Injury assessment and scoring after administration of adipose-derived mesenchymal stem cell and exosomes

Histopathological examination revealed marked differences in lung tissue injury scores among the experimental groups (Table 3). The mean total lung injury score was 3 in the control group and increased to 9.12 following oleic acid administration, representing an increase of 204% compared with the control group and confirming the successful induction of ALI (*p* ≤ 0.05; Table 3, Figs. 6–9).

**Table 3:** Histopathological lung injury scores and gene expression levels of interleukin (IL)-6, IL-10, and vascular endothelial growth factor (VEGF) across experimental groups (mean ± standard deviation).

	Control (n = 5)	OA (n = 5)	ASCT (n = 5)	EXT (n = 5)	*p* -value
TLIS	2.99 ± 0.34	9.12 ± 0.16^a^	5.63 ± 0.42^a b^	7.19 ± 0.32^a b c^	0.000
IL-6	2.12 ± 1.44	5.35 ± 4.43	14.31 ± 7.57	7.69 ± 5.13	0.426
IL-10	6.65 ± 4.76	15.42 ± 8.16	28.29 ± 13.64	33.17 ± 16.40	0.341
VEGF	1.29 ± 0.34	0.92 ± 0.86	6.16 ± 10.90	3.31 ± 1.48	0.424

Control: control group; OA: oleic acid group; ASCT: adipose stem cell treatment group; EXT: exosome treatment group;

a *p* ≤ 0.05 compared with the control group;

b *p* ≤ 0.05 compared with the OA group;

c *p* ≤ 0.05 compared with the ASCT group;

TLIS: total lung ınjury score; IL: ınterleukin; VEGF: vascular endothelial growth factor.

Source: Elaborated by the authors.

**Figure 6:**
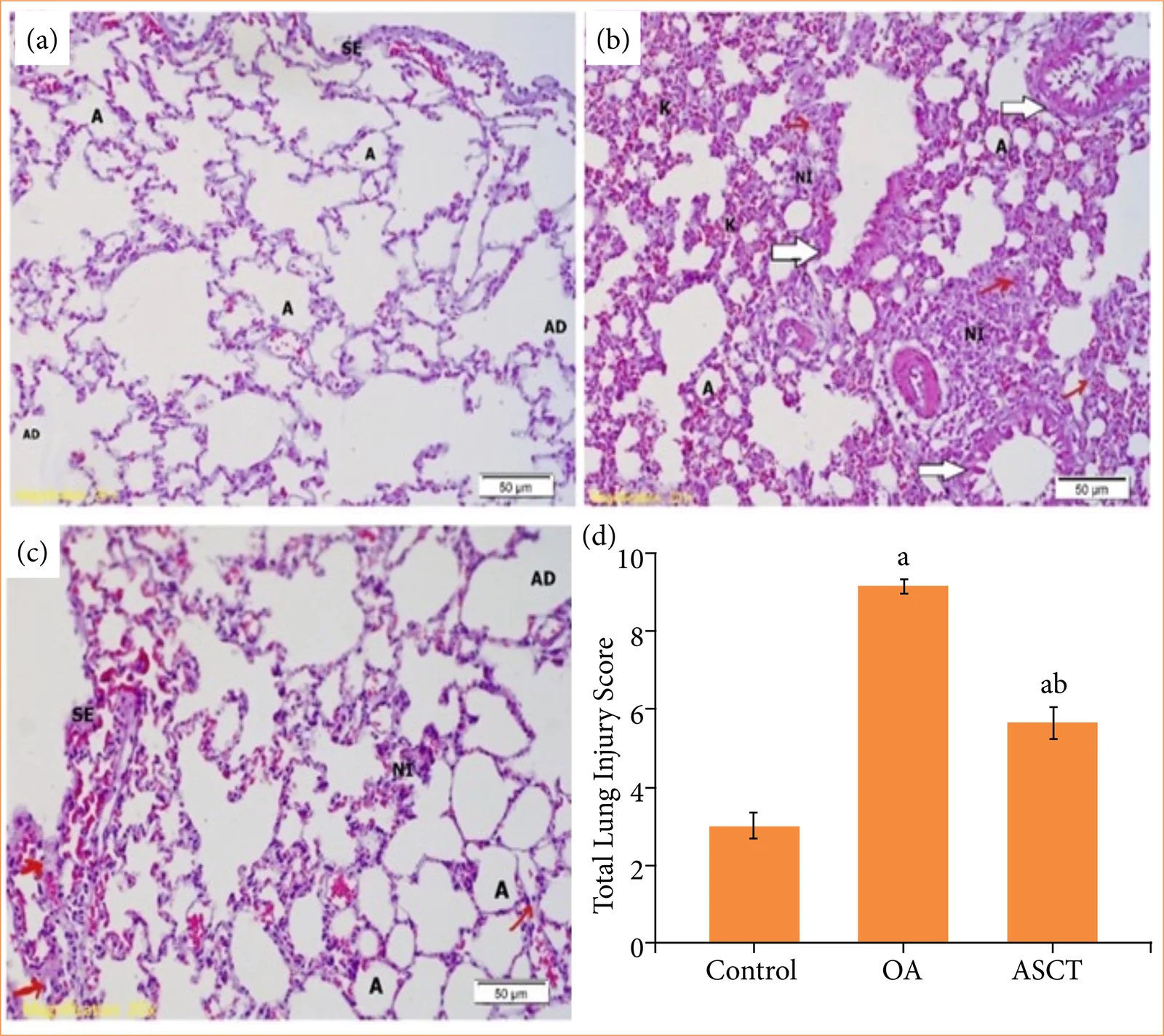
Histopathological analysis of lung tissue following the administration of adipose-derived mesenchymal stem cells in rats with OA-induced acute lung injury along with graphical representation of total lung injury scores. Representative images include the (a) control group, (b) the OA group, and (c) the ASCT group (hematoxylin and eosin staining, 20× magnification). Key histopathological features include alveolus (A), alveolar duct (AD), bleeding (K), neutrophil infiltration (NI), respiratory epithelium (SE), terminal bronchiole (white arrow), and edema (red arrow). (D) Quantitative comparison of injury scores among the experimental groups.

OA: oleic acid; ASCT: adipose stem cell treatment; ^a^ *p* ≤ 0.05 compared with the control group; ^b^ *p* ≤ 0.05 compared with the OA group. Source: Elaborated by the authors.

**Figure 7:**
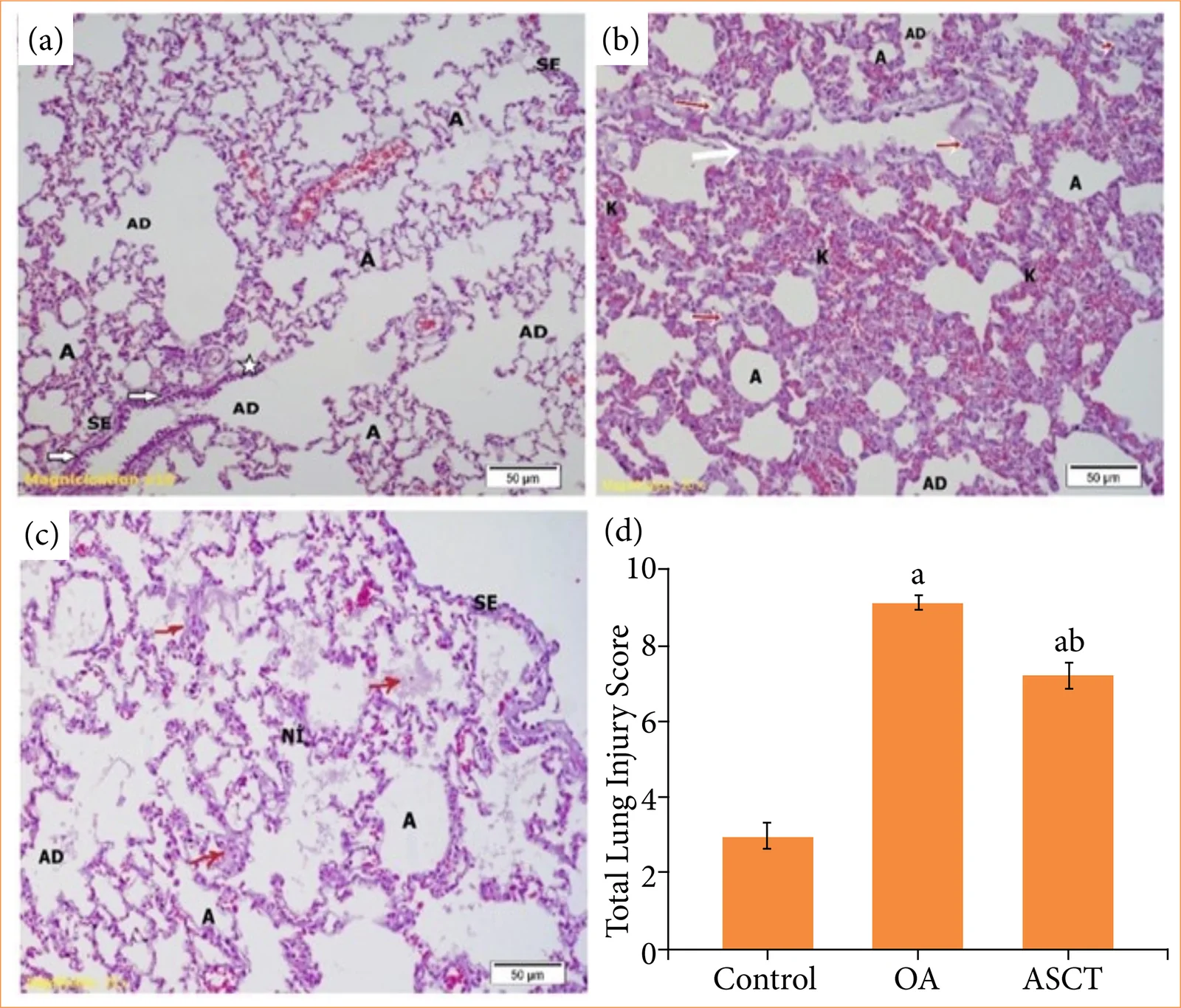
Histopathological analysis of lung tissue following administration of exosomes in rats with OA-induced acute lung injury accompanied by a graphical representation of total lung injury scores. Representative images include the (a) control group, (b) the OA group, and (c) the EXT group (hematoxylin and eosin staining, 20× magnification). (d) Quantitative comparison of lung injury scores across the groups. Histopathological features observed include alveolus (A), alveolar duct (AD), bleeding (K), neutrophil infiltration (NI), respiratory epithelium (SE), terminal bronchiole (white arrow), and edema (red arrow).

EXT: exosome treatment; OA: oleic acid; ^a^ *p* ≤ 0.05 compared with the control group; ^b^ *p* ≤ 0.05 compared with the OA group. Source: Elaborated by the authors.

**Figure 8:**
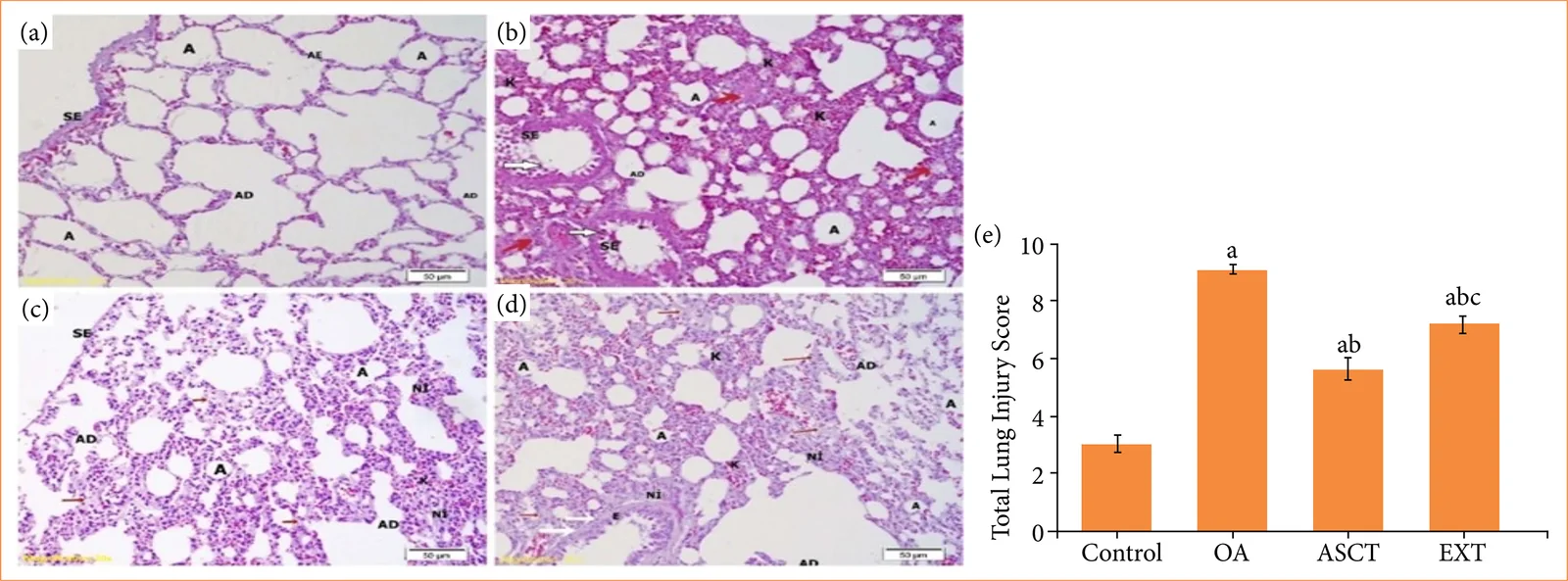
Histopathological evaluation of lung tissue and total lung injury scores in rats with OA-induced acute lung injury following treatment with adipose-derived mesenchymal stem cells or exosomes. Representative images include (a) the control group, (b) the OA group, (c) the ASCT treatment group (OA+ASCT), and (d) the EXT group with OA (hematoxylin and eosin staining, 20× magnification). (e) The total lung injury scores across all groups. Histopathological features observed include alveolus (A), alveolar duct (AD), bleeding (K), neutrophil infiltration (NI), respiratory epithelium (SE), terminal bronchiole (white arrow), and edema (red arrow).

ASCT: adipose stem cell treatment; EXT: exosome treatment; OA: oleic acid; ^a^ *p* ≤ 0.05 compared with the control group; ^b^ *p* ≤ 0.05 compared with the OA group; ^c^ *p* ≤ 0.05 compared with the ASCT group. Source: Elaborated by the authors.

**Figure 9:**
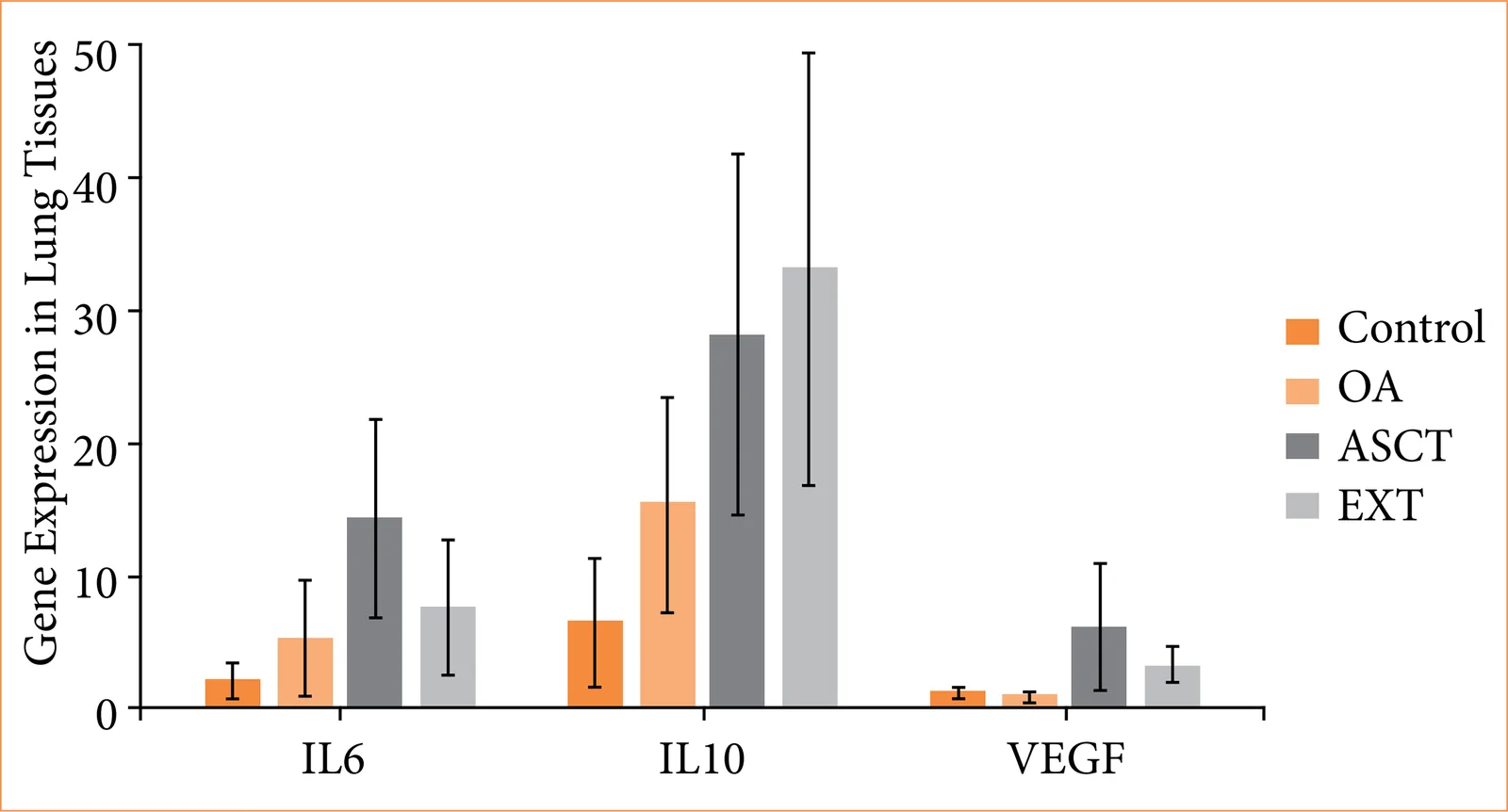
Relative mRNA expression levels of IL-6, IL-10, and VEGF in lung tissues of experimental groups. mRNA expression was evaluated using quantitative real-time polymerase chain reaction, and results were normalized to glyceraldehyde 3-phosphate dehydrogenase (GAPDH) as the endogenous control. No significant differences were observed for IL-6, IL-10, or VEGF expression levels between groups.

ASCT: adipose stem cell treatment; EXT: exosome treatment; OA: oleic acid; IL: interleukin; VEGF: vascular endothelial growth factor. Source: Elaborated by the authors.

Treatment with ADMSCs (ASCT group) reduced the mean total injury score to 5.64, corresponding to a reduction of 38.2% compared with the OA group (*p* ≤ 0.05; Fig. 8, Table 3). Similarly, the EXT showed a reduction in the mean total injury score to 7.19, representing a reduction of 21.1% relative to the OA group (*p* ≤ 0.05; Fig. 8, Table 3). Thus, both treatments were associated with a measurable reduction in histopathological injury compared with the untreated OA group.

The mean total injury score in the ASCT group was 21.7% lower than that in the EXT group, and this difference was statistically significant (*p* ≤ 0.05; Fig. 8, Table 3). These quantitative findings were consistent with the morphological observations. The ASCT group showed greater preservation of lung architecture, characterized by reduced alveolar wall thickening, diminished inflammatory cell infiltration, and preservation of alveolar spaces. In the EXT group, partial morphological recovery was observed, but areas of hemorrhage, edema, and inflammatory infiltration remained evident. Collectively, the quantitative histopathological scores demonstrate a reduction of 38.2% in total lung injury following ADMSC treatment and a reduction of 21.1% following exosome treatment relative to the OA group, providing a quantitative measure of the extent of histopathological improvement (Fig. 8 and Table 3).

### Quantitative real-time polymerase chain reaction analysis of interleukin (IL)-6, IL-10, and vascular endothelial growth factor

qRT-PCR analysis revealed no significant differences in IL-6, IL-10, and VEGF levels among the experimental groups (Fig. 9, Table 3). Although IL-6 expression appeared elevated in the ASCT group and IL-10 levels were numerically higher in both ASCT and EXT groups compared with control and OA groups, these differences were not significant (*p* = 0.426 for IL-6, *p* = 0.341 for IL-10; Table 3). Similarly, VEGF expression increased following ADMSC and exosome treatment, although the differences were not significant (*p* = 0.424; Table 3). These findings indicated that while ADMSC and exosome therapies can influence molecular markers associated with inflammation and angiogenesis, the changes in mRNA expression were not significant under the experimental conditions applied in this study.

## Discussion

Recent studies have highlighted the regenerative potential of extracellular vesicles, particularly exosomes, in various models of lung injury. However, the comparative efficacy of mesenchymal stem cell-based therapies and their secreted products remains under investigation. In the present study, we evaluated the regenerative effects of ADMSCs and their isolated exosomes in an OA-induced ALI Wistar albino rat model. Both treatments exerted protective effects on injured lung tissue, while ADMSCs demonstrated slightly superior histopathological improvement.

Characterization analyses including flow cytometry, SEM, DLS, and zeta potential measurements confirmed successful isolation of ADMSC-derived exosomes with physicochemical properties consistent with previous reports and MISEV2023 recommendations^16-22^.

Histopathological analysis demonstrated that both treatment groups exhibited reduced pulmonary edema, hemorrhage, and inflammatory cell infiltration compared with the OA-only group. These findings are in agreement with previous studies demonstrating the protective effects of mesenchymal stem cells and their derivatives in both experimental ALI models and clinical ARDS settings^23,24^. Jung et al.^5^ reported that human ADMSCs reduced inflammatory injury in an lipopolysaccharide-induced ALI model, whereas Mizuta et al.^16^ demonstrated that ADMSC administration markedly attenuated lung hemorrhage and endothelial cell apoptosis.

Although both therapies improved lung architecture, ADMSCs produced slightly greater histopathological recovery. This difference may be associated with the biological properties of viable stem cells. ADMSCs are capable of responding dynamically to the injured microenvironment and secreting a broad spectrum of cytokines, chemokines, growth factors, and extracellular vesicles that collectively contribute to tissue repair^25,26^. In addition, specialized intercellular communication mediated by ADMSC-derived exosomes, along with extracellular matrix remodeling, may further enhance the overall regenerative activity^27^.

The therapeutic effects of ADMSCs may be explained by their interaction with the inflammatory and vascular microenvironment generated during ALI. OA-induced lung injury is associated with epithelial and endothelial damage, disruption of the alveolar-capillary barrier, increased vascular permeability, and inflammatory cell recruitment. In this setting, ADMSCs may act predominantly through paracrine and immunomodulatory mechanisms rather than direct replacement of damaged cells. Their secretion of cytokines, chemokines, growth factors, and extracellular vesicles may modulate inflammatory responses, support endothelial and epithelial recovery, and promote tissue repair^25-27^. The ability of viable ADMSCs to continuously respond to local tissue signals may partly explain the slightly greater histopathological improvement observed in the ADMSC group.

In contrast, exosomes mainly exert their effects through intercellular signaling mediated by their molecular cargo, including miRNAs, proteins, and lipids. Although exosomes demonstrated clear regenerative effects in our model, their activity may be limited by factors such as fixed cargo composition, biodistribution, immune clearance, and cellular uptake efficiency^28,29^. Nevertheless, previous studies have shown that exosomes can modulate oxidative stress, mitochondrial function, and survival pathways in lung injury models, supporting the regenerative findings observed in the present study^30,31^.

Exosomes may therefore reproduce part of the therapeutic activity of ADMSCs through cell-free intercellular communication. Their molecular cargo can be transferred to recipient cells and may modulate inflammation, oxidative stress, mitochondrial function, apoptosis, and cellular survival^30,31^. These effects may contribute to the recovery of alveolar epithelial and endothelial cells and restoration of pulmonary homeostasis. However, unlike viable ADMSCs, isolated exosomes cannot dynamically adapt their secretory response to changes in the injured microenvironment, which may partly account for the modest difference in histopathological recovery between the two treatment groups.

Post-treatment mRNA analysis of IL-6, IL-10, and VEGF at 24 hours yielded no statistically significant differences between groups. However, both ADMSC and exosome groups exhibited numerically elevated IL-10 and VEGF levels compared with the OA group, suggesting a trend toward anti-inflammatory and pro-regenerative signaling. These findings align with previous literature highlighting the dynamic role of ADMSCs in modulating IL-6, IL-10, and VEGF pathways in ALI environments^32,33^. This lack of significance likely reflects the highly transient nature of cytokine responses, which characteristically peak and normalize within narrow timeframes^34^. Furthermore, inherent biological variability, limited sample size, and tissue heterogeneity may have obscured subtle transcriptional changes.

The absence of statistically significant differences in IL-6, IL-10, and VEGF expression at 24 hours should therefore be interpreted cautiously. The numerical increase in IL-10 and VEGF observed in the treatment groups may be consistent with an early reparative response, but the present study cannot establish a direct mechanistic relationship because gene expression was assessed at a single time point.

Of note, the regenerative mechanisms of ADMSCs and their exosomes might differ at the molecular level. While ADMSCs predominantly modulate inflammation via secreted cytokines and direct interaction with immune cells^35^, exosomes could confer protection via alternative mechanisms, such as by enhancing mitochondrial bioenergetics and antioxidant defenses in alveolar macrophages and endothelial cells^27,30^. These distinct pathways underscore the need for further mechanistic investigations using single-cell transcriptomics, proteomics, and advanced imaging techniques to trace cell and exosome biodistribution *in vivo*.

Taken together, these findings suggest that the therapeutic effects of ADMSCs and their exosomes are likely mediated by multiple interconnected processes rather than a single signaling pathway, including modulation of inflammation, regulation of cellular stress, preservation of epithelial and endothelial integrity, and promotion of tissue repair. However, these mechanisms were not directly investigated in the present study and should therefore be confirmed in future mechanistic studies.

Several limitations should be acknowledged. mRNA expression analysis was performed at a single timepoint, which may not fully reflect transient or delayed cytokine responses during tissue repair. In addition, sample size and biological variability may have limited the statistical power to detect subtle transcriptional differences. Although histopathological improvement was evident, the semi-quantitative nature of histological scoring may introduce subjectivity, and future studies incorporating quantitative functional analyses could provide a more comprehensive evaluation of therapeutic efficacy. Furthermore, the therapeutic effects were evaluated over a short observation period, and the persistence of the observed histopathological and molecular responses remains unknown. Future studies incorporating multiple time points and longer follow-up periods are needed to determine whether these early beneficial effects are sustained and whether ADMSCs and their exosomes differ in the durability of their therapeutic effects.

## Conclusion

Both ADMSCs and their exosomes demonstrated regenerative effects in OA-induced ALI, supporting their therapeutic potential in lung injury. Although ADMSCs showed slightly greater histopathological improvement, exosomes may represent a promising cell-free alternative for future translational applications. The observed effects may involve coordinated paracrine, immunomodulatory, and cell-free intercellular communication mechanisms affecting inflammation, tissue integrity, and repair. Further studies are needed to clarify the underlying mechanisms and optimize therapeutic strategies for clinical use. Longitudinal studies incorporating multiple molecular and functional endpoints will be necessary to determine the durability of these therapeutic responses.

## Data Availability

The data will be available upon request.
